# Patient-Provider Distance in Medicare Outpatient Oncology Telehealth Versus Office Visits, 2021‐2023: Cross-Sectional Claims Analysis

**DOI:** 10.2196/95370

**Published:** 2026-10-23

**Authors:** Lingbo Liu, Tracy Onega, Erika L Moen, Anna N A Tosteson, Rebecca E Smith, Qianfei Wang, Lauren Cowan, Fahui Wang

**Affiliations:** 1 Thrust of Urban Governance and Design, Society Hub, The Hong Kong University of Science and Technology (Guangzhou), Guangzhou, Guangdong, China; 2 Center for Geographic Analysis, Harvard University, Cambridge, MA, United States; 3 Huntsman Cancer Institute, University of Utah, Salt Lake City, UT, United States; 4 Department of Population Health Sciences, University of Utah, Salt Lake City, UT, United States; 5 Department of Biomedical Data Science, Geisel School of Medicine at Dartmouth, Lebanon, NH, United States; 6 The Dartmouth Institute for Health Policy and Clinical Practice, Geisel School of Medicine at Dartmouth, Lebanon, NH, United States; 7 Dartmouth Cancer Center, Geisel School of Medicine at Dartmouth, Lebanon, NH, United States; 8 Department of Geography, University of Hong Kong, 10F, The Jockey Club Tower, Centennial Campus, Pokfulam RoadHong Kong, China (Hong Kong), 852 391-02256

**Keywords:** telehealth, telemedicine, teleoncology, cancer care, Medicare, geographic access, travel burden, rural health, health equity, digital health

## Abstract

In a cross-sectional analysis of traditional Medicare Fee-For-Service beneficiaries aged 66 years or older with outpatient oncology-related encounters, we found that telehealth modestly extended patient-provider geographic reach but largely operated within existing office-based catchments and preserved rural-urban distance gradients. Equitable telehealth policy should target patients with high travel burden while supporting digital access, navigation, and cross-jurisdiction continuity.

## Introduction

For patients with cancer, repeated outpatient visits can impose substantial time, travel, and financial burdens [1,2]. Telehealth is relevant for selected oncology functions, including follow-up, symptom management, medication review, survivorship care planning, and care coordination, but many cancer services still require in-person diagnosis, imaging, infusion, radiation, procedures, or surgery [3,4]. Therefore, telehealth should not be understood as making oncology care distance-free. Instead, an important policy question is whether virtual encounters extend the geographic relationship between patients and oncology providers beyond existing office-based catchments. This question is also equity relevant because telehealth benefits depend on digital infrastructure, patient support, and regulatory contexts, including cross-state care and licensure [5-7]. In this study, patient-provider distance for telehealth represents geographic separation and potential service reach, not physical travel for the virtual encounter. We compared patient-provider distance for telehealth vs office visits in Medicare outpatient oncology care.

## Methods

### Analyses

We analyzed traditional Medicare Fee-For-Service claims in the Centers for Medicare & Medicaid Services (CMS) Virtual Research Data Center and Chronic Conditions Warehouse, a secure environment for approved Medicare claims research. The cohort included beneficiaries aged 66 years or older with outpatient oncology-related encounters for breast, colon, rectal, endometrial, lung, or prostate cancer during 2021 to 2023. Office visits served as the in-person comparator. Telehealth and office visits were mutually exclusive encounter types defined using claims-based telehealth indicators, eligible outpatient visit codes, modifiers, and place-of-service codes. We calculated great-circle distance between beneficiary and provider zip code centroids; this zip-level measure approximates patient-provider geography and, for telehealth, service reach rather than travel. Rurality was assigned using zip-to-RUCA (rural-urban commuting area) crosswalks (UA: urban; LRC: large rural city/town; SRT: small rural town; ISR: isolated small rural town); US Census region was assigned by beneficiary state. We summarized medians, IQRs, cumulative distributions, and d80, the distance containing 80% of visits. Encounters missing beneficiary or provider zip codes were excluded. Estimates are visit weighted.

### Ethical Considerations

This study was determined exempt by the Huntsman Cancer Institute because it involved secondary analysis of existing Medicare claims data. The data were accessed through the CMS Virtual Research Data Center and Chronic Conditions Warehouse under required data use agreements and were analyzed within the secure CMS research environment. Only deidentified, cell size–compliant aggregate outputs were released from the secure environment. Informed consent was waived.

## Results

Median patient-provider distance and dispersion increased with rurality for office and telehealth visits (Figure 1A). Telehealth distance distributions closely tracked those of office visits across all RUCA strata, indicating modest catchment expansion rather than a distinct geography of care. Median distances (miles) for office vs telehealth visits were 11.2 vs 12.1 in UA, 24.5 vs 26.8 in LRC, 35.6 vs 38.2 in SRT, and 42.1 vs 45.3 in ISR.

**Figure 1.**
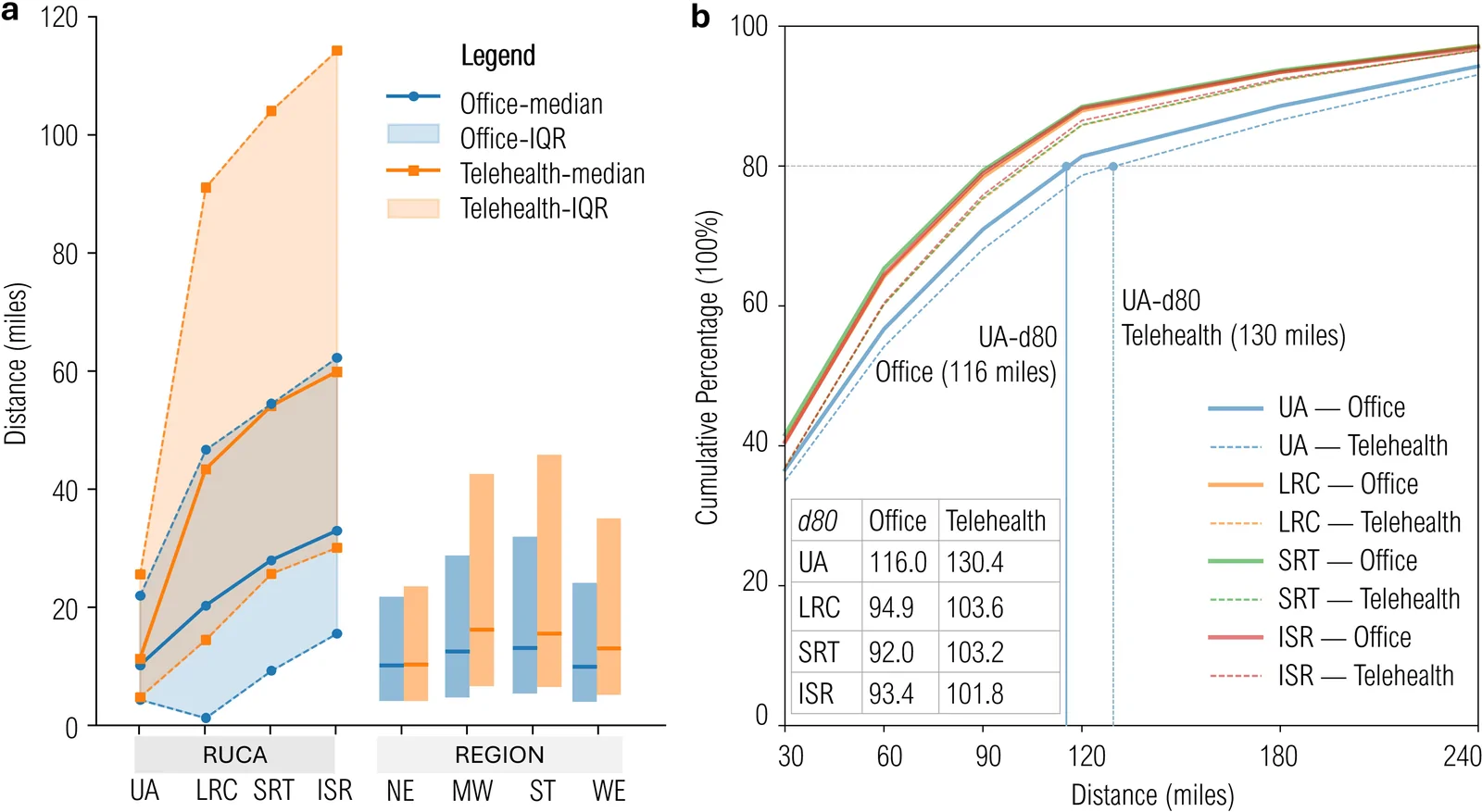
Distance patterns of telehealth vs office visits across rurality and region. (A) Median patient-provider distance (lines) and IQR (Q1-Q3; shaded) for office visits and telehealth visits by RUCA (rural-urban commuting area) category and by US Census region; (B) cumulative distribution of visit distance (miles) by RUCA category for office visits (solid) and telehealth visits (dashed). Inset table reports d80 (miles), the distance covering 80% of visits, for each RUCA stratum. ISR: isolated small rural town; LRC: large rural city/town; MW: Midwest; NE: Northeast; SRT: small rural town; ST: South; UA: urban area; WE: West.

Differences were more evident in the upper tail (Figure 1B). At d80, telehealth extended reach by approximately 8 to 14 miles across RUCA strata. In UA, d80 increased from 116.0 miles (office) to 130.4 miles (telehealth). In LRC, d80 increased from 94.9 to 103.6 miles; in SRT, from 92.0 to 103.2 miles; and in ISR, from 93.4 to 101.8 miles.

Distances also varied by US Census region (Figure 1A). For office visits, median (IQR) distance was 9.8 (4.0 to 21.7) miles in the Northeast, 12.8 (4.7 to 28.7) miles in the Midwest, 13.5 (5.3 to 32.0) miles in the South, and 10.1 (3.9 to 24.0) miles in the West. Corresponding telehealth distances were 10.6 (4.0 to 23.5) miles, 16.4 (6.5 to 42.6) miles, 15.8 (6.3 to 45.9) miles, and 13.3 (5.0 to 35.0) miles, respectively. Across regions, telehealth medians were modestly higher (0.8 to 3.6 miles), and dispersion was broader, but telehealth distances remained close to office distances within each region.

## Discussion

### Principal Findings

In this national Medicare outpatient oncology cohort, telehealth distances largely tracked office visits, with modest outward shifts concentrated in the upper tail. These distances represent patient-provider geography, not physical travel. Telehealth therefore appeared to modestly extend existing oncology networks rather than transform outpatient cancer care, consistent with a hybrid model in which virtual encounters supplement facility-based care that requires physical presence.

The concentration of differences in the upper tail suggests that telehealth may be most relevant for patients who already travel farther, including those in rural areas and those connected to regional specialty centers. At the same time, persistent rural-urban gradients indicate that telehealth alone does not eliminate structural sources of travel burden, including workforce maldistribution, regionalization of specialty services, and uneven digital infrastructure. Equity-relevant evaluation should therefore consider whether telehealth reduces high-burden travel among those most affected and whether gains are equitably realized given digital divides and variation in access to support.

This study has limitations. Distances were measured as straight-line great-circle miles rather than network travel time, which may understate burden in areas with sparse road networks or geographic barriers. Claims data may misclassify visit modality, and unmeasured confounding by case mix, severity, and patient preference may influence observed distributions. Future work should assess patient-level longitudinal trajectories, distinguish substitution from supplementation of in-person care, and evaluate whether telehealth-associated changes in travel burden relate to timeliness, continuity, and outcomes, particularly for populations at risk of digital exclusion.

### Public Health Implications

Telehealth policy should align with how oncology care is delivered in practice. Because observed expansion is modest and concentrated in the long-distance tail, telehealth may be most beneficial as a targeted tool for patients facing higher travel burden rather than as a universal replacement for local care. Rural access and digital equity require broadband affordability, device access, navigation and language support, clinically appropriate coverage options, and licensure or network policies that support continuity across jurisdictions. Monitoring should track travel burden, missed appointments, and time to treatment.

## Abbreviations

CMS: Centers for Medicare & Medicaid Services
d80: distance containing 80% of visits
ISR: isolated small rural town
LRC: large rural city/town
RUCA: rural-urban commuting area
SRT: small rural town
UA: urban area
